# Proteomics Analysis for Identification of Potential Cell Signaling Pathways and Protein Targets of Actions of Atractylodin and β-Eudesmol Against Cholangiocarcinoma

**DOI:** 10.31557/APJCP.2020.21.3.621

**Published:** 2020-03

**Authors:** Kanawut Kotawong, Wanna Chaijaroenkul, Sittiruk Roytrakul, Narumon Phaonakrop, Kesara Na-Bangchang

**Affiliations:** 1 *Chulabhorn International College of Medicine, Thammasat University, Paholyothin Road, Klonglung, *; 2 *Center for Genetic Engineering and Biotechnology (BIOTEC), National Science and Technology Development Agency, Pathumthani Thailand. *

**Keywords:** Atractylodin, β-eudesmol, cholangiocarcinoma, proteomics, signaling pathway

## Abstract

**Objective:**

The study aimed to identify potential cell signaling pathways and protein targets of actions of atractylodin and β-eudesmol in cholangiocarcinoma, the two active compounds isolated from *Atracylodes lancea* using proteomics approach.

**Method:**

The cholangiocarcinoma cell line, CL-6, was treated with each compound for 3 and 6 hours, and the proteins from both intra- and extracellular components were extracted. LC-MS/MS was applied following the separation of the extract proteins by SDS-PAGE and digestion with trypsin. Signaling pathways and protein expression were analyzed by MASCOT and STITCH software.

**Results:**

A total of 4,323 and 4,318 proteins were identified from intra- and extracellular components, respectively. Six and 4 intracellular proteins were linked with the signaling pathways (apoptosis, cell cycle control, and PI3K-AKT) of atractylodin and β-eudesmol, respectively. Four and 3 extracellular proteins were linked with the signaling pathways (NF-κB and PI3K-AKT) of atractylodin and β-eudesmol, respectively.

**Conclusion:**

In conclusion, a total of 17 proteins associated with four cell signaling pathways that could be potential molecular targets of anticholangiocarcinoma action of atractylodin and β-eudesmol were identified through the application of proteomics approach.

## Introduction

Cholangiocarcinoma is the cancer of bile duct arising from epithelial cells and develops along the biliary tree. Classification of cholangiocarcinoma based on anatomical location, the microscopic pattern of growth, microscopic feature, and origin cell (Cardinale et al., 2013). Intra- and extrahepatic cholangiocarcinoma are common types. One important risk factor is the consumption of improperly cooked cyprinoid fishes which contain the liver fluke *Opisthorchis viverrini* (Thamavit et al., 1994; Sripa et al., 2007). Clinical efficacy of the standard chemotherapeutic drugs 5-fluorouracil (5-FU), cisplatin, and gemcitabine given as single drugs or combinations remain unsatisfactory. The discovery and development of effective alternative drugs for control of this cancer are urgently needed. The rhizome extract of *Atractylodes lancea* (Thunb) DC. as well as its major constituents atractylodin and β-eudesmol have been shown to be potential candidates from a series of non-clinical investigation by our research group (Na-Bangchang and Plengsuriyakarn, 2017). All have been identified to exert promising anticancer activity against cholangiocarcinoma both in vitro and in vivo. The anticancer activity of atractylodin has been demonstrated to be linked with inhibition of the interleukin-6 secretion in HMC-1 via MAPKs pathway (Chae et al., 2016). Moreover, gastric emptying could be promoted through ghrelin receptor by activation of atractylodin (Bai et al., 2017). On the other hand, β-eudesmol has been reported to suppress angiogenesis, cell proliferation, and growth of cancer cells (Tsuneki et al., 2005; Ma et al., 2008). Despite this available information on anticancer activity including possible molecular targets of both active compounds, their potential targets of action and signaling pathways in cholangiocarcinoma remain unclear. The study aimed to identify potential cell signaling pathways and protein targets of actions of atractylodin and β-eudesmol using proteomics approach.

## Materials and Methods

*Cell culture*

The CL-6 cell line initially isolated from a patient with advanced-stage cholangiocarcinoma was kindly provided by Associate Professor Dr. Adisak Wongkajornsilp, Faculty of Medicine, Siriraj Hospital, Mahidol University, Thailand. The cancer cell was grown in RPMI 1640 (Gibco, NY, USA) medium, 10% (v/v) heated fetal bovine serum (FBS: Gibco, NY, USA), and 100 IU/ml of anti-biotic-antimycotic solution (Gibco, NY, USA) and maintained at 37^o^C in 5% CO_2_ atmosphere with 95% humidity (HERA CELL 150i, Thermo scientific, MA, USA).

*Sample preparations*

The CL-6 was cultured in a 25 cm^2^ flask (10^6^ cells/flask) and treated with atractylodin (40 µg/ml) or β-eudesmol (40 µg/ml) for 3 and 6 hours. Proteins from both intra- and extracellular components were extracted from each sample for further proteomics analysis. The cell suspension was separated from the attached cells through aspiration. For the intracellular proteins, the treated cell from the cell culture was collected and washed with cold PBS, followed by cell lysis with SDS (0.5%), and centrifugation at 800 xg at 4°C for 10 minutes. Finally, the supernatant was transferred into a new tube and protein concentration was measured the concentration by Lowry method (Lowry et al., 1951). For the extracellular proteins, cell supernatant proteins were precipitated using acetone (-20ºC), and incubated at -20ºC overnight. The sample was centrifuged at 13,000 xg for 15 minutes and the supernatant was discarded. The protein pellet was dissolved with SDS (0.5%) and protein concentration was measured by the Lowry method (Lowry et al., 1951).

*Separation of protein by SDS-PAGE*

The intra- and extracellular proteins extracted were separated on SDS-PAGE according to their molecular weights under denaturing conditions. Briefly, each sample (30 µg protein) was mixed with 2× sample electrophoresis buffer (0.125 M Tris-HCl pH 6.8, 4% SDS, 20% v/v glycerol, 0.2 M DTT, and 0.02% bromophenol blue), heated at 95°C for 5 minutes, and incubated on ice for 2 minutes before loaded into the SDS-PAGE. The proteins were separated with 4% SDS-PAGE as the stacking part and 12.5% SDS-PAGE as the protein separation part. A constant current of 20 mA was applied for 80 minutes to separate proteins that migrated from cathode (+) to anode (-). The Coomassie brilliant blue r-250 was used for protein staining. The stained samples in the gel were divided into 19 fragments by using the six standard protein bands (LMW-SDS Marker Kit, GE Healthcare, UK) as landmarks for cutting. 

*Protein digestion*

Each gel fragment was transferred into a 96-well plate, washed twice with sterile water (5 minutes each), incubated in 100% acetonitrile at room temperature (25^o^C) for 5 minutes, and air-dried at room temperature for 5-10 minutes. The proteins were mixed with denaturing buffer I (10 mM dithiothreitol and 10 mM ammonium bicarbonate) for 1 hour, followed by carbamidomethyl buffer (100 mM iodoacetamide and 10 mM ammonium bicarbonate) for additional 1 hour in a dark room. After digestion with trypsin (10 ng trypsin in a mixture of 50% acetonitrile and 10 mM ammonium bicarbonate) for 20 minutes at 25^o^C, the proteins in the gel were extracted three times with acetonitrile (30%). The acetonitrile extracts were pooled and dried at 40°C (3-4 hours or overnight) and stored at -80°C until use.

*LC-MS/MS analysis*

The dried sample in a 96-well plate was dissolved with 0.1% formic acid and centrifuged at 3,000x g for 5 minutes. The supernatant was transferred into the insert tube and protein content identified by Ultimate 3,000 LC system (Dionex) coupled to ESI-Ion Trap MS (HCT ultra PTM Discovery System, Bruker Daltonik) with electrospray at a flow rate of 20 μl/minute to μ-precolumn (Monolithic Trap Column, 200 μm i.d. x 5 mm). The proteins were separated on a nano column (Monolithic Nano Column, 100 μm i.d. x 5 cm) with a solvent gradient (solvent A: H_2_O, 0.1% formic acid; solvent B: 50% H_2_O, 50% ACN, and 0.1% formic acid) running at a flow rate of 1 µL/minute for 20 minutes.

*Analysis of LC-MS/MS data*

The LC-MS/MS data were analyzed using the DeCyder MSTM (Amersham Bioscience AB, Uppsala, Sweden), and the proteins were identified by the MASCOT program (http:// www.matrixscience.com). The identification of the signaling pathways of these proteins was performed using STITCH software (http://stitch.embl.de/).

*Cell apoptosis and cell cycle analysis*

The apoptosis and cell cycle arrest data were obtained from previously published data (Kotawong et al., 2018). The cell apoptosis was identified by FITC Annexin V Apoptosis Detection Kit I and analyzed by flow cytometer. And cell cycle analysis was performed using flow cytometry with propidium iodide (PI) stained (BD FACSVerse™ flow cytometer, BD, USA).

## Results

*Proteomic analysis*

**Figure 1:**
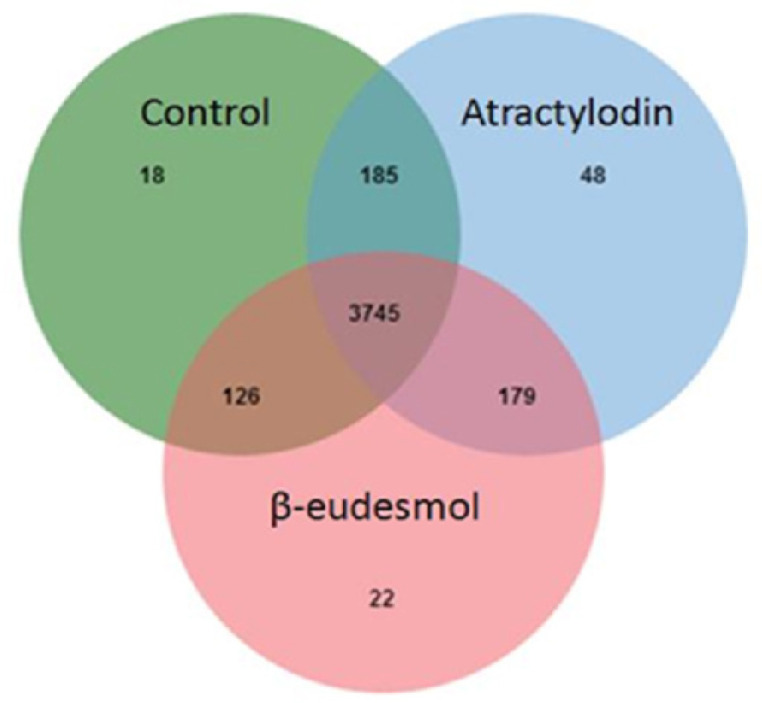
Venn Diagram of the Proteins Identified from the Intracellular Cell Component after Exposure of CL-6 Cells to Atractylodin, β-Eudesmol, and Control

**Figure 2:**
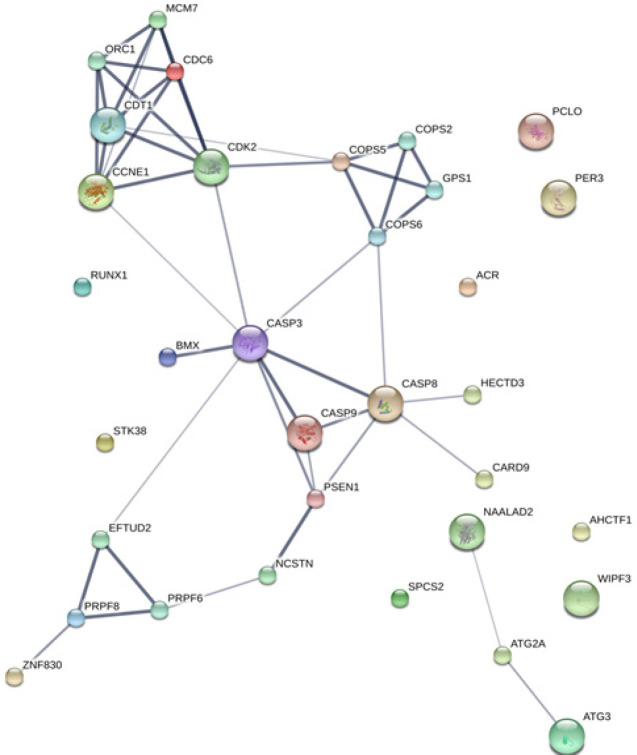
The Analysis by STITCH Software, of the Interactions among 48 Proteins that were Found only from Intracellular Cell Component of Atractylodin-Treated CL-6 Cells

**Figure 3:**
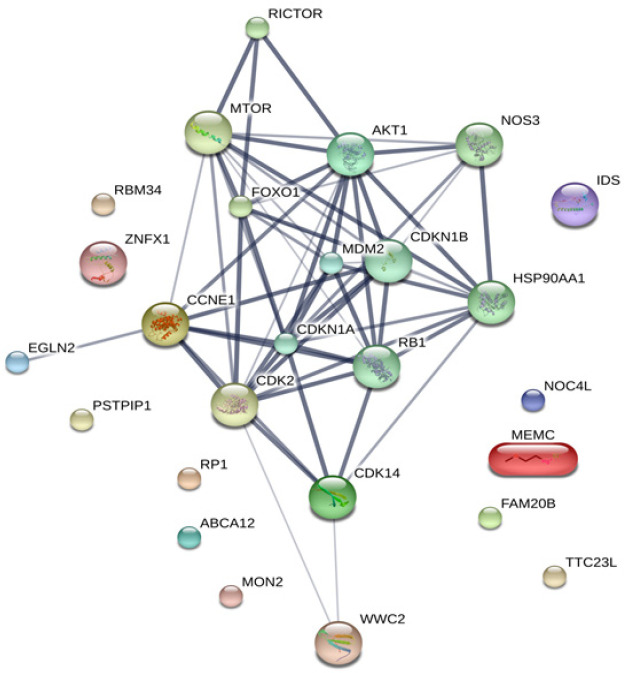
The Analysis by STITCH Software, of the Interactions among 48 Proteins that were Found only from Intracellular Cell Component of β-Eudesmol -Treated CL-6 Cells

The MASCOT analysis identified 4,323 and 4,318 proteins from the intra- and extracellular components of CL-6 cells, respectively, following exposure to atractylodin and β-eudesmol. For the intracellular proteins, 18, 48, and 22 proteins were only found in control (untreated), atractylodin-treated and β-eudesmol-treated CL-6 cell, respectively (Figure 1). The involvement of these proteins in different cell signaling pathways was further analyzed by STITCH software. Results showed that 19 of 48 and 15 of 22 proteins from atracylodin-treated and β-eudesmol-treated CL-6 cells, respectively, were matched with the STITCH database. Seven out of 19 proteins from atractylodin-treated cells were identified to be involved in the two cell signaling pathways, i.e., apoptosis (5 proteins) and cell cycle control (2 proteins) (Table 1). The five proteins identified in the apoptosis pathway included MX non-receptor tyrosine kinase protein (BMX), caspase recruitment domain family, member 9 (CARD9), HECT domain-containing E3 ubiquitin-protein ligase 3 (HECTD3), and presenilin1 (PSEN1). The BMX, HECTD3 and CARD9, and PSEN1 proteins were identified as components of caspase 3, caspase 8 (extrinsic pathway), and caspase 9 (intrinsic pathway), respectively. The two proteins identified in the cell cycle control were cell division cycle 6 homolog (CDC6) and COP9 constitutive photomorphogenic homolog subunit 5 (COPS5) proteins (Figure 2). Both proteins control cell division at the G1 phase of the cell cycle. Four out of 15 proteins from β-eudesmol-treated cells were identified to be involved in the two signaling pathways, i.e., cell cycle control (3 proteins) and PI3K-AKT (1 protein) pathways (Figure 3) (Table 1). The three proteins identified in the cell cycle pathway were WW and C2 domain containing 2 (WWC2), cyclin-dependent kinase 14 (CDK14), and egl nine homolog 2 (EGLN2). These proteins were identified to link with cell cycle control via cyclin E1 (CCNE1), cyclin-dependent kinase 2 (CDK2), and retinoblastoma 1 (RB1) at G1 phase. For the PI3K-AKT pathway, only v-akt murine thymoma viral oncogene homolog 1 (AKT1) protein which is involved in cell proliferation and survival was identified.

**Figure 4:**
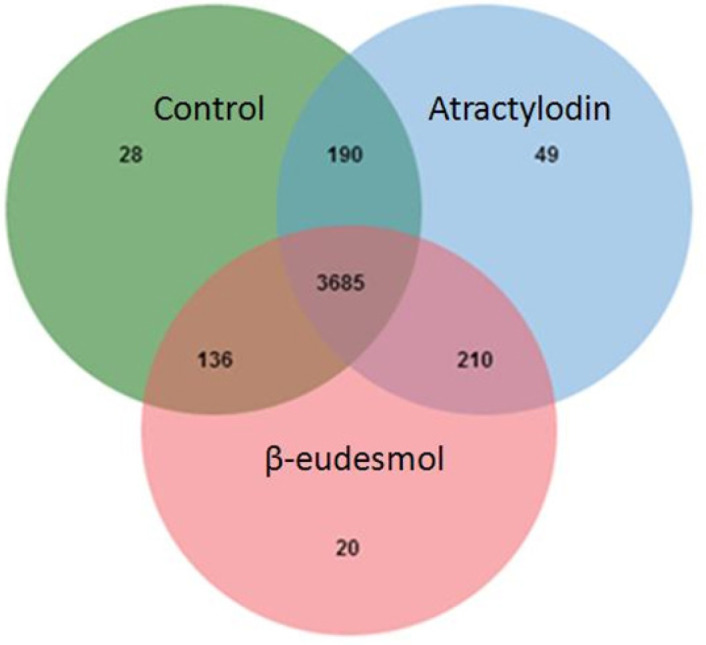
Venn Diagram of the Proteins Identified from the Extracellular Cell Component after Exposure of CL-6 Cells to Atractylodin, β-Eudesmol, and control

**Figure 5:**
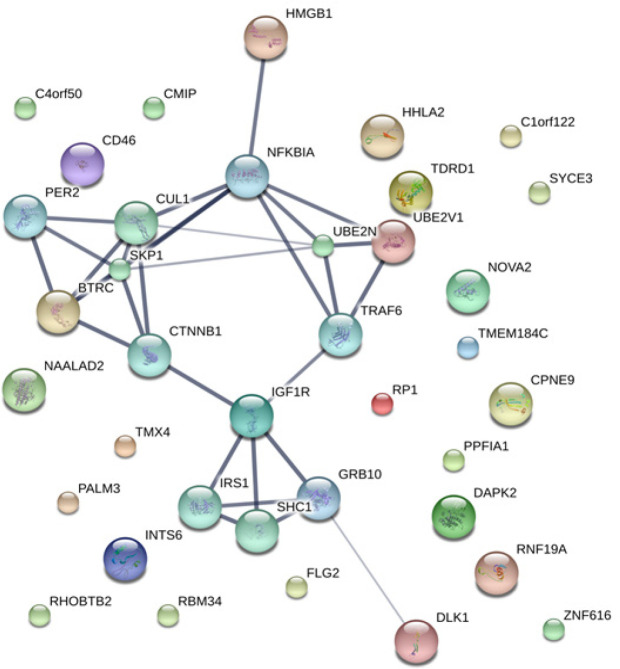
The Analysis by STITCH Software, of the Interactions among 48 Proteins that were Found only from Extracellular Cell Component of Atractylodin-Treated CL-6 Cells

**Figure 6:**
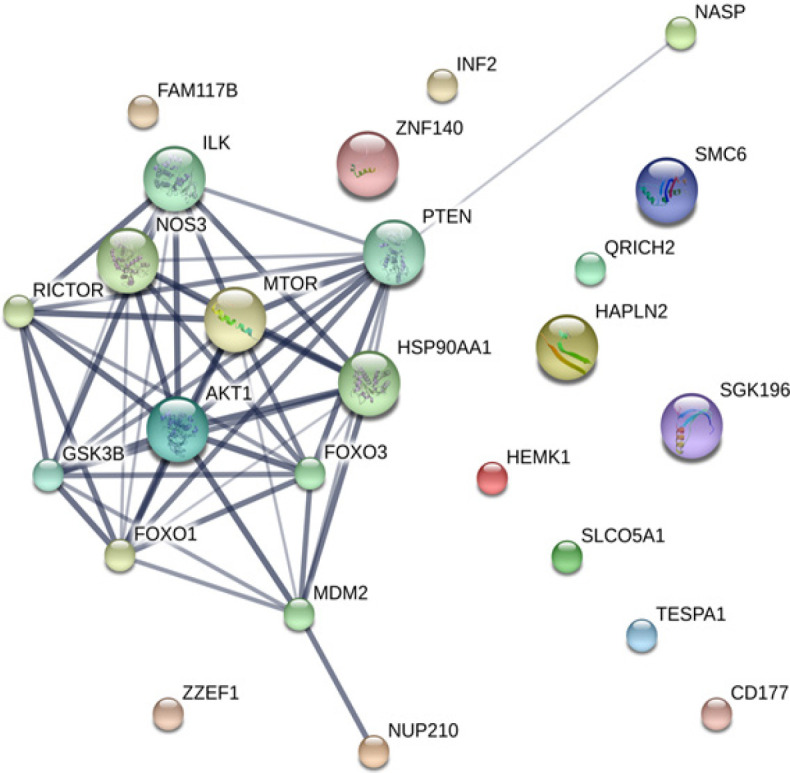
The Analysis by STITCH Software, of the Interactions among 48 Proteins that were Found only from Extracellular Cell Component of β-Eudesmol -Treated CL-6 Cells

Concerning the extracellular proteins, 28, 49, and 20 proteins were only found in control (untreated), atractylodin-treated and β-eudesmol-treated CL-6 cell, respectively (Figure 4). Only 27 of 49 and 15 of 20 proteins from atractylodin-treated (Figure 5) and β-eudesmol-treated (Figure 6), respectively, were matched with the STITCH database. Four proteins from atractylodin-treated cells were identified to link with the NF-κB pathway, i.e., high mobility group box 1 (HMGB1), ubiquitin-conjugating enzyme E2 variant 1 (UBE2V1), thioredoxin domain containing 5 (TXNDC5), and paralemmin 3 (PALM3) (Table 1). Moreover, cells the toll-like receptor was identified as the receptor of NF-κB signaling. The signaling pathway identified for the β-eudesmol-treated cell was PI3K-AKT pathway which involved three proteins, i.e., v-akt murine thymoma viral oncogene homolog 1 (AKT1), nuclear autoantigenic sperm protein (NASP), and nucleoporin 210 kDa (NUP210) (Table 1). This PI3K-AKT pathway was identified to be regulated by NASP and NUP210 proteins via phosphatase and tensin homolog (PTEN) and mouse double minute 2 (MDM2), respectively. 

*Cell apoptosis and cell cycle analysis*

From the proteomic data, both atractylodin and β-eudesmol -treated cells were involved in apoptosis and cell cycle control. This information was confirmed by the previous report of the apoptosis and cell cycle analysis data. Both atractylodin and β-eudesmol could induce cell apoptosis, which depended on time and concentration. In consistence with proteomic data, both compounds induced cancer cell arrest at the G1 phase by time dependence and concentration dependence (Kotawong et al., 2018).

**Table 1:** Correlation between Protein and Signaling Pathway

Stages	Protein localizations	Compounds	Signaling pathways	Protein names
Early	Intracellular	Atractylodin	Apoptosis	Included MX non-receptor tyrosine kinase protein (BMX) Presenilin1 (PSEN1)
			Cell cycle	Cell division cycle 6 homolog (CDC6) COP9 constitutive photomorphogenic homolog subunit 5 (COPS5) Lipin 3 (LPIN3) Zinc finger protein 830 (ZNF830)
			Autophagy	Autophagy related 2A (ATG2A) Autophagy related 3 (ATG3) N-acetylated alpha-linked acidic dipeptidase 2 (NAALAD2)
		β-eudesmol	PI3K-AKT	Akt murine thymoma viral oncogene homolog 1 (AKT1)
			Cell cycle	Cyclin-dependent kinase 14 (CDK14) Egl nine homolog 2 (EGLN2) WW and C2 domain containing 2 (WWC2)
	Extracellular	Atractylodin	Cell cycle	Beta-transducin repeat containing E3 ubiquitin protein ligase(BTRC) Delta-like 1 (Drosophila)(DLL1) Integrator complex subunit 6(INTS6) Thioredoxin domain containing 5 (TXNDC5) Ubiquitin-conjugating enzyme E2 variant 1(UBE2V1)
		β-eudesmol	Autophagy	Plectin (PLEC)
			Migration	High mobility group box 1(HMGB1)
			PI3K-AKT	Akt murine thymoma viral oncogene homolog 1 (AKT1)
			Cell cycle	Nuclear autoantigenic sperm protein (histone-binding) (NASP)
Late	Intracellular	Atractylodin	Cell cycle	BRCA1 associated RING domain 1(BARD1)
			Angiogenesis	Insulin-like growth factor 1 receptor ( IGF1R)
			Apoptosis	Flightless I homolog (FLII)
		β-eudesmol	Cell cycle	E3 ubiquitin-protein ligase (ARIH1)
			Autophagy	Signal transducer and activator of transcription 1(STAT1)
	Extracellular	Atractylodin	Apoptosis	Rho related BTB domain containing 2 (RHOBTB2)
				Trio Rho guanine nucleotide exchange factor (TRIO)
			Cell cycle	AT hook containing transcription factor 1(AHCTF1)
				Centromere protein T(CENPT)
				Kinesin family member 3A(KIF3A)
			Cell cycle	Serine/threonine kinase 38 (STK38)
		β-eudesmol	Immune respond	Signal toll-like receptor 9 (TLR9)

**Table 2:** Comparison of the Functions of Proteins Found in the Current and Other Studies

Protein names	Current study		Other studies	
	Cell type	Biological regulations	Cell type	Biological regulations
FLII	CCA cell	Cell apoptosis	Squamous cell carcinoma	Cell apoptosis (Kopecki et al., 2015)
RHOBTB2	CCA cell	Cell apoptosis	Human thyroid cancer cell line	Cell apoptosis and cell cycle (Wang et al., 2015b)
TRIO	CCA cell	Cell apoptosis	Hepatocellular carcinoma	Cell apoptosis (Wang et al., 2015a)
			Human podocytes	Cell growth (Maier et al., 2018)
BMX	CCA cell	Cell apoptosis	Cervical cancer	Cell cycle (Li et al., 2016; Li et al., 2017)
PSEN1	CCA cell	Cell apoptosis	Gastric cancer	Cell metastasis (Li et al., 2016; Li et al., 2017)
KIF3A	CCA cell	Cell cycle	Benign prostate cell	Cell growth (Liu et al., 2014)
COPS5	CCA cell	Cell cycle	Laryngeal squamous cell carcinoma	Cell apoptosis and cell cycle (Li et al., 2018)
IGF1R	CCA cell	Cell angiogenesis	Prostate cancer	Cell growth and migration (Heidegger et al., 2014)
HMGB1	CCA cell	Cell migration	Osteosarcoma	Cell cycle and invasion (Meng et al., 2014)
			Human lung cancer	Cell migration and invasion (Zuo et al., 2014)
			Gastric cancer	Cell invasion (Zhang et al., 2014)
TLR9	CCA cell	Immune respond	Prostate cancer	Cell migration and invasion (Luo et al., 2015)
AHCTF1	CCA cell	Cell cycle	HeLa cells	Cell cycle (Rasala et al., 2006; Clever et al., 2012)
ATG3	CCA cell	Autophagy	Leukemia cell lines	Cell apoptosis (Zhuang et al., 2016)
STAT1	CCA cell	Autophagy	Mouse embryonic fibroblasts	Cell apoptosis (Townsend et al., 2004)

## Discussion

The proteomics approach was applied in the current study to initially investigate the possible signaling pathways and protein targets involved in the mechanisms of action of the two active principles from *A. lancea *(Thunb) DC., atractylodin and β-eudesmol. The crude ethanolic extract of the rhizomes of this plant, as well as both active compounds, have previously been reported for their potential anticancer activity against cholangiocarcinoma, both in vitro and in vivo (Na-Bangchang and Plengsuriyakarn, 2017). The proteins isolated from intra- and extracellular components of the cholangiocarcinoma cell line (CL-6) following exposure to atractylodin and β-eudesmol were identified by LC-MS/MS. The only proteins which were specific to atractylodin or β-eudesmol were further analyzed by STITCH software for the identification of the cell signaling pathways and target proteins involved. The signaling pathways that involved associated with atractylodin cytotoxic effect against CL-6 cells were shown to be apoptosis, cell cycle, and NF-κB pathways. On the other hand, the signaling pathways associated with β-eudesmol were cell cycle control and PI3K-AKT.

Apoptosis is the programmed cell death that could be found in both normal and abnormal cells. Cell apoptosis can occur during embryogenesis, metamorphosis, and upon exposure of the cells to chemicals and UV light that lead to DNA damage (Roos and Kaina, 2006). The morphological changes of cell apoptosis include cell shrinkage, pyknosis, plasma membrane blebbing, and cell fragmentation (Saraste and Pulkki, 2000). Induction of cell apoptosis involves two main pathways, i.e., intrinsic and extrinsic pathways (Elmore, 2007). For the intrinsic pathway, DNA damage results in cell signaling to Bcl2, Bax, mitochondria, cytochrome C, caspase 9, and caspase 3 via p53 (Loreto and La Rocca, 2014). The extrinsic pathway is associated with activation of the death receptors on the cell surface, resulting in cell signaling to caspase 8 and caspase 3 (Fulda and Debatin, 2006). Bid protein connects the signaling of the intrinsic and extrinsic pathways. Abnormality of apoptosis pathways have been shown to link with cancers (Gobe et al., 2002; Han et al., 2002) as well as several other pathological conditions including Alzheimer (Nikolaev et al., 2009), Parkinson (da Costa and Checler, 2010), rheumatoid arthritis (Eguchi, 2001) and ischemia (Lopez-Neblina et al., 2005). Cancer is the pathological condition of uncontrolled cell growth and apoptosis pathways have been identified as important potential targets for anticancer drug development. Hypericin, the bioactive compound from Hypericum perforatum was shown to induce apoptosis of the breast cancer cell MCF-7 via increasing of p53 and decreasing Bcl2 (Mirmalek et al., 2015). Chalepin from *Ruta angustifolia* was shown to induce lung cancer cell apoptosis through the intrinsic pathway via caspase 9 and 3 (Richardson et al., 2016). For cholangiocarcinoma, cordycepin isolated from fungus (Wang et al., 2017a) and schisandrin B isolated from the fruit of *Schisandra chinensis *(Yang et al., 2016) were demonstrated to activate cell apoptosis through the intrinsic pathway via Bax and caspase 3. 

Cell cycle (cell division), the cell process of increasing cell numbers, consists of four main phases, i.e., G1 (Gap 1), S (Synthesis), G2 (Gap 2), and M (Mitosis) phases (Pucci et al., 2000). Three cell cycle checkpoints (from G1 to S, G2 to M and M to G1) control cell division. The G1 to S phase checkpoint controls cell cycle via specific proteins cyclins (E and D), cyclin-dependent kinases (CDK2, 4 and 6), retinoblastoma protein (RB), and transcription factors (E2F) (Pietenpol and Stewart, 2002). These proteins could be exploited as promising anticancer targets. The sulfated polysaccharide isolated from *Laurencia papillosa *was shown to down-regulate breast cancer cell (MDA-MB-231) division via suppression of cyclin E and D and thus induction of G1-phase cell cycle arrest (Murad et al., 2016). Farnesiferol C isolated from *Ferula asafetida *was found to regulate G1-phase arrest in breast cancer cells (Hasanzadeh et al., 2017). Matrine isolated from *Sophora flavescens* was shown to cause G1-phase arrest in T-cell lymphoblastic leukemia via decreasing of CDKN1A (Tetik Vardarli et al., 2017). For cholangiocarcinoma, G1-phase cell cycle arrest was demonstrated with berberine, an alkaloid from *Berberis vulgaris* and *Berberis aquifolium *via NF-κB and STAT3 pathways (Puthdee et al., 2017). Besides, it also caused cell cycle arrest at the G2 phase via interfering with cyclin B and CDK1 checkpoints (Innocente et al., 1999). Increasing of Cyclin B-CDK1 complex could have driven the cell from G2 to M phase. Inhibition of gastric cancer and nasopharyngeal carcinoma cell growth through induction of G2/M phase arrest was demonstrated with withaferin A, a steroidal lactone from *Withania somnifera* (Kim et al., 2017) and cardamonin, a chalcone from *Cardamom spice* (Li et al., 2017c). The human colon cancer cell cycle arrest at the G2 phase was induced by tetraarsenic hexoxide through increasing p21 and decreasing cyclin B levels (Nagappan et al., 2017). For the last checkpoint at M phase, the mistake of this process can lead to several conditions including Down’s syndrome (Herault and Delabar, 2017), turner’s syndrome (Culen et al., 2017) and acute myeloid leukemia (addition copy of chromosome 8) (Nucifora and Rowley, 1995).

PI3K-AKT pathway is an important signaling pathway that controls cell growth and cell survival. Signal transduction starts once the receptor (receptor tyrosine kinase and epidermal growth factor receptor) is induced upon ligand binding, resulting in activation of PI3K and AKT. AKT controls cell proliferation via p21 and p27 (Xu et al., 2017) and regulates cell apoptosis via MDM2 (Laroche et al., 2017) and Bax (Feng et al., 2017). The phosphatase and tensin homolog deleted on chromosome 10 (PTEN) protein was shown to regulate the PI3K-AKT pathway via inhibition of the transformation of PIP2 to PIP3. The growth of several types of cancer has been reported to be regulated by the PI3K-AKT pathway. Synaptojanin 2 binding protein (SYNJ2BP) is the protein that is highly expressed in breast tumors. The increase in SYNJ2BP expression was shown to promote the degradation of PTEN leading to cancer cell metastasis (Wang et al., 2017b). In gastric cancer, the PI3K-AKT pathway was shown to be induced by netrin-1 (Yin et al., 2017) and phosphatidylethanolamine-binding protein 4 (PEBP4) (Wu et al., 2017b) both of which are proteins commonly found in many cancers. Increasing the levels of these proteins resulted in the enhancement of the invasion and metastasis of gastric cancer. For cholangiocarcinoma, garlicin the active ingredient of garlic was shown to inhibit cancer cell invasion and migration via PI3K/AKT signaling pathway (Xie et al., 2015).

Nuclear factor-κB (NF-κB) is the transcription factor that plays an important role in cell inflammation (Lawrence, 2009), cell proliferation (Wu et al., 2017a) and cell survival (Schweighoffer and Tybulewicz, 2017). This pathway is also triggered by ligand-gated receptor binding on the cell surface. The receptors involved include epidermal growth factor receptor (EGFR) (Saxon et al., 2016) toll-like receptor (TLR) (Sun et al., 2017) and tumor necrosis factor receptor (TNFR) (Lee et al., 2017). Activation of these receptors leads to receptor phosphorylation through IKK complex and transcription of the relevant genes. Ursolic acid from *Ligustrum lucidum* and the chemotherapeutic drug cisplatin, have been shown to inhibit cervical cancer cell growth through interfering with NF-κB signaling pathway via decreasing p65 level (Li et al., 2017a). Xanthohumol from *Humulus lupulus L. *was also shown to inhibit pancreatic cancer cell angiogenesis through interfering with this pathway (Saito et al., 2017). 

These potential molecular targets of atractylodin and β-eudesmol for CCA cells identified in the current study were also reported in previous studies (Table 2). Further study should be performed to confirm the link between the anticholangiocarcinoma activity of atractylodin and β-eudesmol and the expression of the identified proteins involved in apoptosis, cell cycle control, PI3K-AKT, and NF-κB signaling pathways. Moreover, apart from apoptosis, cell cycle control, PI3K-AKT and NF-kB signaling pathways, other pathways and proteins should be further investigated as their possible potential targets of action against human cholangiocarcinoma. 

## Fundings

KK gratefully acknowledges the financial support provided by Thammasat University Research Fund under the TU Research Scholar, Contract No. 02/2561 and Thammasat University Ph.D. program scholarship. This research was funded by Center of Excellence in Pharmacology and Molecular Biology of Malaria and Cholangiocarcinoma of Thammasat University (1/2556), National Research Council of Thailand (NRCT).
